# Occupational Burnout levels in Emergency Medicine–a nationwide study and analysis

**Published:** 2010-08-25

**Authors:** F Popa, R Arafat, VL Purcărea, A Lală, G Bobîrnac

**Affiliations:** * >‘Carol Davila’ University of Medicine and Pharmacy, Bucharest Romania; ** Romanian Ministry of Health, BucharestRomania

## Abstract

Introduction: The specificity of the emergency medical act strongly manifests itself on account of a wide series of psycho–traumatizing factors augmented both by the vulnerable situation of the patient and the paroxysmal state of the act. Also, it has been recognized that the physical solicitation and distress levels are the highest among all medical specialties [1], this being a valuable marker for establishing the quality of the medical act.

Material and Methods: We have surveyed a total of 4725 emergency medical workers with the MBI–HSS instrument, receiving 4693 valid surveys (99.32% response rate). Professional categories included Emergency Department  doctors (M–EMD), ambulance doctors (M–AMB), ED doctors with field work in emergency and resuscitation (including mobile intensive care units and airborne intensive care units) (D–SMU), medical nurses in Emergency Departments (N–EMD), medical nurses in the ambulance service (N–AMB), ED medical nurses with field activity in emergency and resuscitation (N–SMU), ambulance drivers (DRV) and paramedic (EMT). The n values for every category of subjects and percentage of system coverage (Table 3) shows that we have covered an estimated total of 29.94% of the Romanian emergency medical field workers.

Results: MBI–HSS results show a moderate to high level of occupational stress for the surveyed subjects. The average values for the three parameters, corresponding to the entire Romanian emergency medical field were 1.41 for EE, 0.99 for DP and 4.47 for PA (95% CI). Average results stratified by professional category show higher EE average values (v) for the M–SMU (v=2.01, 95%CI) and M–EMD (v=2.21, 95%CI) groups corresponding to higher DP values for the same groups (vM–EMD=1.41 and vM–SMU=1.22, 95%CI). PA values for these groups are below average, corresponding to an increased risk factor for high degrees of burnout. Calculated PA values are 4.30 for the M–EMD group and 4.20 for the M–SMU group.

Conclusions: Of all surveyed groups, our study shows a high risk of burnout consisting of high emotional exhaustion (EE) and high depersonalization (DP) values for Emergency Department doctors, Emergency, and Resuscitation Service doctors (M–SMU). Possible explanations for this might be linked to high patient flow, Emergency Department crowding, long work hours and individual parameters such as coping mechanisms, social development and work environment.

Further research on high–risk groups (M–EMD and M–SMU) has been implemented in the on–going phase Ⅱ of our study.

The specificity of the emergency medical act strongly manifests itself on account of a wide series of psycho–traumatizing factors augmented both by the vulnerable situation of the patient and the paroxysmal state of the act. Also, it has been recognized that the physical solicitation and distress levels are the highest among all medical specialties [1], this being a valuable marker for establishing the quality of the medical act. As studies have continuingly linked physical drainage of the physician with low quality medical services, special measures have been taken to insure a secure work environment for both the practitioner and the patient. Emergency medicine is currently the first to have a limitation of the on–call period to 12 hours (as opposed to 24 for other specialties), and even so, the average ER professional life of emergency medical technicians worldwide is that of four years, similar to that of EM specialists[1]. 

Several risk factors have been shown to have a great impact on physician stress levels, of these, some are specific for medics working on mobile intensive care units and some are general. Social condition of the patient. Interacting with patients of precarious social condition as that of dysfunctional families, low financial grading or extreme conditions that make protocols useless (dangerous social environments) may influence communication between parts and interfere in the decision–making process. The prescribing of long–term treatment on mobile units in developing countries is a present reality, and because of the low medical and social education of the patient, this becomes an important factor if it has to be taken into consideration that a future visit to the hospital for further investigations is unlikely. This being said, situations like this require a fast, technical anamnesis with the purpose of revealing objective clinical aspects, in addition to circumstantial evidence that should complete a global view on the medical situation.  Professional authority becomes, in these cases, fundamental in the development of an efficient medical act. This may go to the point of cutting off any interpersonal relationship between the parts (in the spirit of treating the illness, not the patient), but reestablishing that relationship is mandatory once the medical act or crisis situation has ended. It has been shown that this reestablishment of interpersonal connection is the highpoint marker of the patient long–term compliance to treatment.[2]

## Transfer and counter–transfer specificity

There are three primary sources of negative transfer that the EM practitioners are subjected to: aggressive patients (where sedation becomes mandatory for the protection of the doctor, at the cost of being unable to perform a correct and efficient anamnesis), uncommunicative patients and aggressive next of kin.

The negative counter–transfer is to be avoided in all its manifestations because it will lead to a negative response on behalf of the patient by means of further augmenting a hostile attitude or aggressiveness.[3] Affective neutrality is of upmost importance because in spite of their feedback on the doctor, the patient and next of kin have as primary concern the solving of the acute medical situation, and so the hostile environment will improve proportionally with the improvement of patient wellbeing. This clearly limits the paternalist attitude of the practitioner since he has no prerogative to judge or condemn the patient for his actions or words if these do not intervene in the therapy process.

## Case variability approach

Another factor that can cause chronic distress is the case variability that requires an advanced affective self–control and empathic selectivity on behalf of the EM specialist. Often there is the situation when an ED is packed with patients from variable fields of medicine (geriatrics, pediatrics, trauma care, cardiology etc.) that require stabilizing before being referred to the specific ward. This process requires high efficiency of the medical training but also a well–developed focusing capacity that enable the specialist to have an optimum psychological approach to each one of his patients. Added to the short amount of time that is allowed for the empathic switch, this aspect furthermore contributes to the accumulation of chronic distress. Furthermore the near aspect of referring the patient soon after stabilization depletes the EM specialist of the psychological reward of having the patient cured, fact which, in time, can lead to feelings of uselessness on behalf of the doctor. Post ED consults of the EM specialists have been implemented successfully in the UK and US and studies that try to link the existence of these consults with a lower rate of burnout are in course.

## GOMER patients 

Described as ‘patients that have lost everything that is human in them’, the term GOMER (stands for Get out Of My Emergency Room) [4], refers to elder patients with multiple neurological and psychiatric pathologies (schizophrenia, multiple dementia), or decompensate forms of pathologies that cannot be treated in the ER. The issue of these patients has been repeatedly discussed because of the multitude of problems they impose. They are the main cause of ER crowding, are what is known as ‘old customers’ of certain hospitals and basically arise as an important stress factor both for the doctors and for the other patients, being perpetual attractors of negative counter–transfer.[5]

All this being said, it is mandatory to consent that, still, the final position on these patients is that there is no possibility of denying medical consult or treatment of a patient based on the above mentioned aspects, because of ethical and public judgments. [6]

Most studies linking the specialist's increased professional stress levels with a low quality medical act have a specific reference to emergency medicine. The average percentage given for doctors that have a higher than normal stress level has been an over–time constant in the area of 28%, in comparison to the 18% shown by the general population [7]. What has shown a clear change in the last years is the openness of the doctors to admit the existence of these problems and discuss them with colleagues or take part in group sessions of therapy. [8]

The far most important factor has been established as the lack of sleep on behalf of the doctor. This aspect has recently been given a high level of attention both by researchers and by management branches of medical world. In the US and other countries following US protocols the maximum on–call period for an EM specialist has been dropped to 12 hours, half of that established for all other specialties, in recognition of the increased level of both physical and psychical stress that the doctor is subjected to. This being said, there is still no measurement taken that prohibits the medic to do overtime at private clinics or other hospitals, therefore a lot of controversies have arisen about the exact efficiency of these measurements.[1,9]

Either individual or conjectural, professional stress sources for EM specialists arise from many aspects of their activities. Among the most discussed are self criticizing attitudes, lack of communication, deficiencies in team–work and lack of professional support, deficient financial rewards, lack of personal time and the lack of self esteem caused by a faulty and unfounded hierarchy that places emergency medical specialists at the bottom of the list.

The theories of Adler later enhanced by Mosak [10, 11], define certain typologies that are used as a conceptual support in pre–establishing coping methods (for the purpose of the spotting and preventive eventual negative strategies). This gives very good intelligence to grading burnout risk in medical workers, and has an important approach and applicability for emergency medicine. (Table 1)

**Table 1:** The conceptual classification of individuals according to central themes of their personality, with special refference to the characteristics that are present in emergency medicine and paramedical workers, according to Adler and Mosak

Centered on control	They supress their feelings and spontaneity in order to gain control Fidelity towards order, intelectual reasoning, consistency and attention to detail Perfectionist, they underapreciate others in comparison to theirselfs
Centered on leading	Active, aggresive, energic They feel the need to be in center of attention and to lead others Ambitious and hardly accepting criticism and advice They feel the need to plase everybody aiming for appreciation They dedicate theirselfes totally to the aimed goal and they prove an excessive conscienciosness
Centered on pleasing	They feel the need to plase everybody at any moment, and to be apreciated Extremely sensitive to criticism, they feel emotionally drained when they don't succeed in constantly plase others They analyze people around in order to identify what plases them, and change their attitude according to these evaluations They appreciate theirselfes according to opinions expressed by their
Centered on gaining	They consider life to be cruel to them for refusing them what they consider they deserve They exploit and manipulate their anturaj by intimidation, personal charm or any other means in order to gain what they want They are unstable in their desires and they mannifest that in all aspects of their lifes
‘Martirs’	Self–compassionate, without hope, predisposed to accidents They dedicate theirselfes to their goal and set it in center of all aspects of their life They brag and pride with their suffering and consider theirselfes disadvanteged and underappreciated
‘Oposants’	They contradict any arguments but their own They do not support any cause and are always ready to react They are passive of others demands and opinions, are extremely pesimistic and foind unique and independet solutions to the issues they are
Centered on reasoning	They avoid feelings and affection, they consider that reason can solve any problem disregarding its nature They are very calculated, affraid of their own spontaneity which they supress as much as possible They do not feel confortable in their social surroundings but they hunt for the good appreciation of their anturaj
‘Action hunters’	They hate routine and repettitive actions, they are very affected by boredom and monothony They seek different anturages in which their needs for challenges may be satisfied

These studies grade individuals based on central points of interest in their personalities in one of the following classes: centered on leadership, centered on control, centered on getting, centered on pleasing, martyrs / victimizers, centered on opposition, centered on rationalization and the so called ‘action hunters’.

Studies have shown that this theory might be useful in grading the personality dynamics of EM personnel by establishing that there are some categories, that are prone to intense burnout, and, contrarily, there are certain classes that seem to have a psychological immunity to this syndrome. Therefore, personalities centered on control have a high burnout susceptibility because of their inconstant and spontaneous decision making process (they are the ‘outside the box’ thinkers), as do those centered on pleasing due to the high patient mortality rate in EM. Also, there is a similar but lower risk for the opposing type and for the rationalizers, since there is a contrast between personal psychological needs and the ‘hands on, rapid thinking’ nature of the EM processes.

Personalities centered on getting, leadership and ‘action hunters’ are considered to have certain immunity to burnout symptoms because of their great ambition and strong sense for getting the most out of the moment [12].

## Material and methods

### Demographics

**Graph 1:**
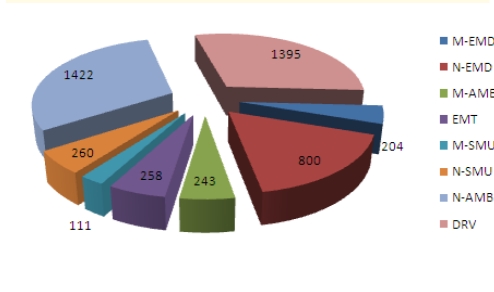
Study n values

**Graph 2:**
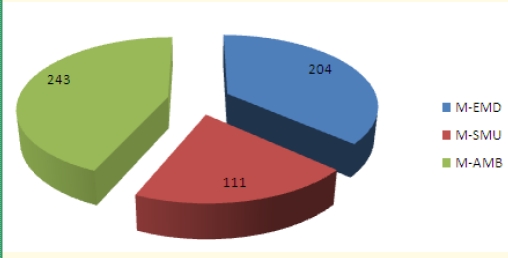
Study n values for MD subjects

**Graph 3:**
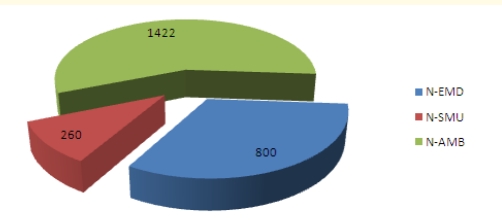
Study n values for medical nurses

We have surveyed a total of 4725 emergency medical workers with the **MBI-HSS** instrument, receiving 4693 valid surveys (99.32% response rate). Professional categories included Emergency Department doctors (**M–EMD**), ambulance doctors (**M–AMB**), ED doctors with field work in emergency and resuscitation (including mobile intensive care units and airborne intensive care units) (**D–SMU**), medical nurses in Emergency Department s (**N–EMD**), medical nurses in the ambulance service (**N–AMB**), ED medical nurses with field activity in emergency and resuscitation (**N–SMU**), ambulance drivers (**DRV**) and paramedic (**EMT**). The n values for every category of subjects and percentage of system coverage (Table 2) shows that we have covered an estimated total of 29.94% of the Romanian emergency medical field workers. Distribution of surveyed subjects according to their professional category (Graph 1) shows higher coverage for ambulance nurses and drivers, in correlation with their prevalence in the system. Distribution of personnel with an MD degree and specialty in Emergency medicine and distribution of medical nurses according to their professional category were taken into account (Graph 2 and Graph 3).

We have measured average burnout levels for every category and for 39 Romanian state districts, as shown in Table 3, aiming for a follow–up study on categories that show a high risk.

**Table 2:** Distributions of subjects by proffessional category and estimated percentage of Romanian emergency medical system coverage. 95% Confidence Limits

	n	Sys%
M–EMD	204	25.79%
N–EMD	800	29.17%
M–AMB	243	34.71%
EMT	258	10.12%
M–SMU	111	45.12%
N–SMU	260	48.12%
N–AMB	1422	28.44%
DRV	1395	18.12%
TOTAL and Average	4693	29.94%

**Table 3:** Average MBI-HSS factor levels for 39 Romanian districts. 95% Confidence Interval

	District	EE factor	PA factor	DP factor
1	Bucharest	1,75	4,22	1,16
2	Provence	1,41	4,47	0,99
3	Brasov	1,76	4,43	1,42
4	Bacau	1,62	4,48	0,99
5	Hunedoara	1,63	4,38	1,15
6	Suceava	1,83	4,37	1,00
7	Teleorman	1,05	4,80	0,70
8	Dambovita	1,50	4,36	0,88
9	Alba	1,07	3,86	1,12
10	Galati	1,81	4,24	1,36
11	Olt	2,07	4,48	1,35
12	Tulcea	0,80	4,69	0,61
13	Dolj	2,33	4,66	1,54
14	Calarasi	1,15	4,55	0,79
15	Cluj	1,65	4,12	1,16
16	Harghita	1,04	4,34	0,77
17	Bihor	1,25	4,64	0,87
18	Mures	1,28	4,48	1,01
19	Satu Mare	1,72	4,51	1,26
20	Buzau	1,18	4,50	0,89
21	Neamt	1,14	4,68	0,75
22	Bistrita–Nasaud	1,34	4,37	0,97
23	Maramures	1,78	4,60	1,04
24	Valcea	1,80	4,66	1,05
25	Ilfov	1,59	4,24	1,64
26	Arges	1,11	4,90	0,75
27	Timisoara	1,07	4,46	0,80
28	Giurgiu	2,27	4,23	1,22
29	Constanta	2,23	4,14	1,65
30	Gorj	1,35	4,88	0,58
31	Covasna	1,17	4,21	0,75
32	Mehedinti	2,04	4,69	1,05
33	Sibiu	1,14	4,67	1,07
34	Salaj	1,42	3,79	1,09
35	Ialomita	1,78	4,52	0,98
36	Botosani	2,22	4,41	1,13
37	Vaslui	1,12	4,48	0,72
38	Prahova	1,54	4,53	0,89
39	Braila	1,23	4,86	0,79
40	Vrancea	1,50	4,31	1,18

### Instrument

The Maslach Burnout Inventory–Human Services Survey MBI–HSS is an instrument designed to assess the three components of the burnout syndrome: emotional exhaustion (EE), depersonalization (DP), and reduced personal accomplishment (PA). There are 22 items, which are divided into three subscales. The items are written in the form of statements about personal feelings or attitudes (e.g., ‘I feel burned out from my work,’ ‘I don't really care what happens to some recipients’). The items are answered in terms of the frequency with which the respondent experiences these feelings, on a 7–point, fully anchored scale (ranging from 0, ‘never’ to 6, ‘every day’). [13]

The reliability coefficients for the MBI–HSS were based on samples that were not used in the item selections to avoid any improper inflation of the reliability estimates. Internal consistency was estimated by Cronbach's coefficient alpha (n=1,316). The reliability coefficients for the subscales were the following: .90 for Emotional Exhaustion, 0.79 for Depersonalization, and 0.71 for Personal Accomplishment. The standard error of measurement for each subscale is as follows: 3.80 for Emotional Exhaustion, 3.16 for Depersonalization, and 3.73 for Personal Accomplishment. [13,14]

Burnout of medical workers has three major components, each one setting a stronger impairment to establishing an efficient doctor–patient relationship. 

The first component is established as accentuated feelings of emotional drainage (EE) and is characteristic for paramedics and emergency medical specialists. This leads to a development of negative psychical experiences manifested outward trough negative counter–transfer that is damaging to the patient. Furthermore, there is a proven link between negative counter–transfer and a high risk of faulty medical decisions and diagnosis [15]. The root of this aspect rests in excessive emotional solicitation due to strong empathic requests of the specialist [16]. In addition, mobile intensive care units and ambulance workers must sometimes carry out their work in tough conditions such as poor lighting, bad weather conditions, negative passer–by interactions, danger environments (fires, floods, chemical disasters etc.) or critical social situations (street fights, armed attacks) that rise even more the psycho–traumatic risk levels of this sort of medical practice. 

The second component (DP) is the tendency to un–individualize the patient for the purpose of downgrading psychological involvement and the third is the tendency to negative auto–evaluation of the specialists which adds stress factors to the process of continuous medical education by augmenting the feeling of hopelessness.

The Personal Accomplishment component assesses feelings of competence and successful achievement in the subject's work with people. In the MBI–HSS, in contrast to the other two subscales, lower mean scores on this subscale correspond to higher degrees of experienced burnout. [13,14]

Analysis of the results was made using Microsoft Excel 2007 (only basic functions) and SPSS software. For the SPSS, one–way analysis of variance (ANOVA) was initially used to test if there were significant differences for burnout components among the categories of subjects. Later, this was supplemented with post–hoc Tukey tests to identify categories exposed to highest burnout risk.

## Results

MBI–HSS results show a moderate to high level of occupational stress for the surveyed subjects. The average values for the three parameters, corresponding to the entire Romanian emergency medical field were 1.41 for EE, 0.99 for DP and 4.47 for PA (95% CI). Average results stratified by professional category (Table 4) show higher EE average values (v) for the M–SMU (v=2.01, 95%CI) and M–EMD (v=2.21, 95%CI) groups corresponding to higher DP values for the same groups (vM–EMD=1.41 and vM–SMU=1.22, 95%CI). PA values for these groups are below average, corresponding to an increased risk factor for high degrees of burnout.

**Table 4:** Average MBI-HSS subscale values, stratified by professional category. 95% CI

	Category	EE	PA	DP
1	M–EMD	2,21	4,30	1,41
2	N–EMD	1,65	4,47	1,10
3	M–AMB	1,78	4,55	0,95
4	EMT	0,63	5,01	0,46
5	M–SMU	2,01	4,20	1,22
6	N–SMU	1,91	4,52	1,35
7	N–AMB	1,36	4,59	0,86
8	DRV	1,13	4,31	1,01

Calculated PA values are 4.30 for the M–EMD group and 4.20 for the M–SMU group. Ambulance MDs higher than the average for the EE factor (vEE=1.78), but PA and DP values correspond to a lower average burnout (vPA=4.55, vDP=0.95, 95% CI).

Results for surveyed nurses show values higher than average for the EE and DP factor in the N–SMU (vEE=1.91, vDP=1.35 ) and N-EMD (vEE=1.65, vDP=1.10), but higher PA values for these groups 4.52 for the N–SMU versus 4.47 for N–EMD. In contrast, the N–AMB group has better–than–average values for all parameters (EE=1.36, DP=0.86, PA=4.59), showing lower degrees of burnout for this category of personnel. (Table 4)

The best–recorded values are for paramedics (EE=0.63, DP=0.46, PA=5.01), with the significance of this detail discussed in the conclusions section. Also, good parameters were found for ambulance drivers (EE=1.13, DP=1.01, PA=4.31).

**Graph 4:**
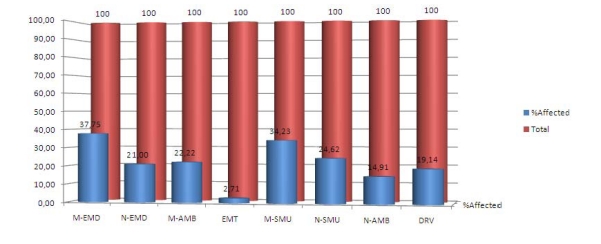
Percentage of respondents with at least one risk value

The percentage of responders that scored an average value over 3.00 for EE and DP or under 3.00 for the PA factor, values correlated with a moderate to high degree of burn–out and set as predictive risk factors (Graph 4) shows a highest values for the M–EMD (37.75%), M–SMU (34.23%) and M–AMB (22.22%), showing the true ‘burden bearers’ of the emergency medical field.  

High–risk average values (EE>4, DP>4 and PA<2) were found in a total of 15 respondents (Table 5).

ANOVA was used to test the differences of burnout components among the 7 categories of study subjects. The burnout scores for all three components (EE, PA, DP) differed significantly (Table 6):

1. F (EE) (7, 4877) = 73,095, p < .0001.
2. F (PA) (7, 4877) = 16,848, p < .0001
3. F (DP) (7, 4877) = 31,137, p < .0001

**Table 5:** Percentage of high–risk responders stratified by professional category. 95% CI

	Category	n	%
1	M–EMD	3	1,47
2	N–EMD	4	0,50
3	M–AMB	0	0,00
4	EMT	0	0,00
5	M–SMU	3	2,70
6	N–SMU	0	0,00
7	N–AMB	4	0,28
8	DRV	1	0,07

**Table 6:** ANOVA results

		Sum of squares	DF	Mean square	F	Sig.
EE	Between groups	583,740	7	83,391	73,095	,000
	Within groups	5336,935	4678	1,141		
	Total	5920,674	4685			
PA	Between groups	145,664	7	20,809	16,848	,000
	Within groups	5776,728	4677	1,235		
	Total	5922,392	4684			
DP	Between groups	181,692	7	25,956	31,137	,000
	Within groups	3897,936	4676	,834		
	Total	4079,628	4683			

To highlight the amount of differences between each pair of categories, Tukey post–hoc comparisons were compiled:

1. For **EE**:The M–EMD group has the highest EE (M = 2,21, 95% CI [2,03–2,39] and its scores are similar only to the M–SMU scores (M = 2,01, 95%CI [1,79–2,24], otherwise it differs from  all other groups.The EMT group is significantly different from all others (M = 0,63, 95%CI [.56 –.70]) with lowest EE scores, meaning the highest protection from emotional exhaustion. (Table 7).
2. For **PA**:EMTs have the highest PA scores (M = .99, 95%CI [.88–1,1] and differs significantly from all other groups. (Table 8)
3. For **DP**The M–EMD and N–SMU groups have the highest DP scores (M = 1,41, 95%CI [1,24–1,57] and M = 1,35, 95% CI [1,21 – 1,49]), making them the most exposed groups to depersonalization. (Table 9).Again, the EMT group has the lowest DP scores (M = .46, 95% CI [.39–.53]), and differs significantly from all other groups.

**Table 7:** Distribution of EE, by groups (Tukey post–hoc comparisons) (p <.05)

M–EMD and M–SMU
N–EMD, M–AMB, N–SMU
N–AMB, DRV
EMT

**Table 8:** Distribution of risk PA, by groups (Tukey post-hoc comparisons) (p <.05)

M–EMD, N–EMD, M–SMU, DRV
EMT

**Table 9:** Distribution of DP, by groups (Tukey post–hoc comparisons) (p <.05)

M–EMD, N–SMU
N–EMD, M–SMU, N–SMU
M–AMB, N–AMB
EMT

## Discussion

A large percentage of emergency physicians experienced a high level of emotional exhaustion (37,75%) which is the core manifestation of burnout, studies show this value to be slight higher than other nationwide values [17, 18], but the prevalence of burnout among hospital staff alone (average value of 12,78%) is higher than that reported in other studies [19], therefore the situation needs further analysis with follow–up detailed studies. Personal accomplishment levels are high, in concordance with other detailed studies [20], but higher PA values were found for the nursing staff (average PA=4.52) than the MD degree staff (average PA= 4.42), and this may be due to a more efficient work hour limits, as the M–EMD and M–SMU MD's have a 24j shift, while nursing staff has a 12h shift, this leading to higher sleep deprivation issues[21], which may intensify the level of burnout by increasing EE and lowering PA. The negative effects of this problem are compounded by job stress and traditional methods of scheduling work shifts [22]. Depersonalization was low for all studied groups, with no remarkable difference, but higher than average values were found for the M-EMD and M-SMU groups. This may be correlated with the higher patient flow and crowded Emergency Department s, which represents a key problem in the development of burnout among physicians.[22, 23, 24] Also, the most stressful aspects of work are reported as being access block, dealing with management, insufficient staffing, workload pressures and staff supervision [24], issues that are consistently present only in the –ED groups, since ambulance staff have a more solitary, tight independent and auto–managing way in which they work, and low interactions with the rest of the patient–care system.

Nurses in all studied groups experienced lower emotional exhaustion than their fellow doctors in all groups, contrary to other studies. [25] We asses this to the better work program nurses have, as well as with lower responsibility in patient care, since the Romanian medical system awards most of the responsibility and consequential problems of the patient care to the doctor. Also, depersonalization was low among the nursing staff, with slight differences between the N–AMB and N–SMU groups. Higher values for the N–SMU groups may be due to the complexity of the medical cases this group handles (major acute trauma, major acute cardiac issues, fieldwork in major car accidents, high–terrain interventions), in comparison with the N–AMB group, which handles low–degree emergencies. Overall levels of burnout were similar or lower on all subscales than previous studies [26, 27], but further research is needed to correlate the differences in burnout levels observed between different nursing groups. Studies have correlated similar levels of emotional exhaustion or overall burnout with more consistent issues such as suicidal ideation (especially among ambulance staff) [28] and alcohol abuse [29].

The paramedical staff shows the lowest degrees of burnout on all three subscales. Paramedics are fire fighters with paramedical training working as first responder teams, using paramedical staffed ambulances. This being a new workforce in the Romanian medical system, they have an average career time of 3 years, and the average staff age is lower than in all surveyed categories–further research is needed to correlate these factors. Burnout levels are lower than those found in other studies [30] and the results might be correlated with the fact that paramedical staff is army–enrolled and must pass periodical psychological examinations. Psychological examination is not mandatory for any other of the surveyed staff. Personal accomplishment for the paramedical staff was the highest of all groups (vPA=5.01) which shows a high job satisfaction, strongly correlated with self–efficacy and spiritual well–being [31, 32], highly important factors for quality of life improvement. 

Ambulance drivers show low emotional exhaustion and low depersonalization scores, attributed to low interaction with patients, but also show a low score on the personal accomplishment scale. The proven stressing and high–risk factor work environment [33, 34, 35] does not show itself in the MBI–HSS results, and needs further research aiming to detect and improve pending issues among this group.

## Conclusions

Among all surveyed groups, our study shows the highest risk of burnout (expressed as high emotional exhaustion (EE) and depersonalization (DP) scores) for doctors working in Emergency Departments and Emergency and Resuscitation Services (M–SMU). In the average risk group we have placed medical nurses from all studied services (N–SMU, N–AMB and N–EMD), although N–SMU had high DP values and most nurses groups had low personal accomplishment scores. Lowest risk for all burnout components was noticed for the Paramedics (EMT) group. M–EMD, M–SMU and EMT groups differed significantly from all the other studied groups, as shown by ANOVA analysis. 

Possible explanations for these findings might be linked to different organization of work within the studied services (e.g. aspects as patient flow, crowding, work hours) but also to different individual characteristics, such as coping mechanisms or social development. 

Study limitations include the risk of conformism for some subjects (due to the vertical top–down distribution of questionnaires), the impossibility to ensure the randomization of the paramedics (EMT) group (this being the only group that is submitted to regular psychological testing and selection) and the lack of a pilot study. Still, the high n–value (n = 4725) and response rate (rr = 99.32%) may compensate these limitations.

Further research on high risk groups (M–EMD and M–SMU) has been already implemented in the on–going phase 2 of our study, in order to establishing causal factors, coping mechanisms and possible repercussions of occupational burnout. We feel that this kind of extensive research of burnout is highly desirable to ensure the proper psychological management of emergency medical workers, to raise their job satisfaction and to increase both the worker's quality of life and the quality of the medical act.

## References

[1] Martini S, Cynthia L (2006). Comparison of Burnout Among Medical Residents Before and After the Implementation of Work Hours Limits. Acad. Psychiatry.

[2] Luban–Plozza B (2002). Dimensiunea psihosocială a practicii medicale.

[3] Parsons C (1969). Sistemul Social.

[4] Shem S, Samuel T (1978). House Of God.

[5] Berliner H (1994). Patient Dumping–No One Wins and We All Lose. American Journal of Public Health.

[6] Winnebago (1973). Mercy Medical Center Congress Preceedings.

[7] Firth–Cozens J (1999). Stress in health professionals: psychological and organizational causes and interventions.

[8] Firth–Cozens J (2001). Interventions to improve physicians' wellbeing and patient care. Soc Sci Med.

[9] U.S.A. Congress (2001). Patient and Physician Safety and Protection Act of 2001.

[10] Adler A (1956). The individual psychology of Alfred Adler.

[11] Mosak HH (1968). The interrelatedness of the neuroses through central themes. Journal of Individual Psychology.

[12] Kosinski F (2000). Journal of Employment Counseling.

[13] Maslach C, Jackson SE (1996). Maslach Burnout Inventory.

[14] Zalaquet C, Wood RJ (1997). Evaluating Stress.

[15] Hammer J, Mathews J (1987). Annals of Emergency Medicine.

[16] Miller K, Birkholt M (1995). Empathy and burnout in human service work: An extension of a communication model. Communication Research.

[17] Kuhn G, Goldberg R (2009). Tolerance for uncertainty, burnout, and satisfaction with the career of emergency medicine. Ann Emerg. Med..

[18] Cydulka RK, Korte R (2008). Career satisfaction in emergency medicine: the ABEM Longitudinal Study of Emergency Physicians. Ann Emerg Med.

[19] Palmer–Morales Y, Prince–Vélez R (2007). Prevalence of burnout syndrome in nurses in 2 Mexican hospitals. Enferm Clin..

[20] Gandini BJ, Paulini SS (2006). The professional wearing down or syndrome of welfare labor stress (‘burnout’) among health professionals in the city of Cordoba. Rev Fac Cien Med Univ Nac Cordoba.

[21] Nelson D (2007). Prevention and treatment of sleep deprivation among emergency physicians. Pediatr Emerg Care.

[22] Cubrilo–Turek M, Urek R (2006). Burnout syndrome–assessment of a stressful job among intensive care staff. Coll Antropol..

[23] Rondeau KV, Francescutti LH (2005). Emergency department overcrowding: the impact of resource scarcity on physician job satisfaction. J Healthc Manag..

[24] Crook HD, Taylor DM (2004). Workplace factors leading to planned reduction of clinical work among emergency physicians. Emerg Med Australas.

[25] Gillespie M, Melby V (2003). Burnout among nursing staff in accident and emergency and acute medicine: a comparative study. J Clin Nurs..

[26] Bell RB, Davison M (2002). A first survey. Measuring burnout in emergency medicine physician assistants. JAAPA.

[27] Hu YC, Chen JC (2010). Nurses' perception of nursing workforce and its impact on the managerial outcomes in emergency departments. J Clin Nurs.

[28] Sterud T, Hem E (2008). Suicidal ideation and suicide attempts in a nationwide sample of operational Norwegian ambulance personnel. J OEDp Health.

[29] Sterud T, Hem E (2007). OEDpational stress and alcohol use: a study of two nationwide samples of operational police and ambulance, personnel in Norway. J Stud Alcohol Drugs.

[30] Nirel N, Goldwag R (2008). Stress, work overload, burnout, and satisfaction among paramedics in Israel. Prehosp Disaster Med..

[31] Duggleby W, Cooper D (2009). Hope, self–efficacy, spiritual well–being and job satisfaction. J Adv Nurs..

[32] Prati G, Pietrantoni L (2010). Self–efficacy moderates the relationship between stress appraisal and quality of life among rescue workers. Anxiety Stress Coping.

[33] Takeda E (2007). OEDpational accidents among ambulance drivers in the emergency relief. Rev Lat Am Enfermagem.

[34] Ray AM, Kupas DF (2007). Comparison of 
rural and urban ambulance crashes in Pennsylvania. Prehosp Emerg Care.

[35] Berry S (2006). Don't let your kids grow up to be ambulance drivers. JEMS.

